# Joint modeling strategy for using electronic medical records data to build machine learning models: an example of intracerebral hemorrhage

**DOI:** 10.1186/s12911-022-02018-x

**Published:** 2022-10-25

**Authors:** Jianxiang Tang, Xiaoyu Wang, Hongli Wan, Chunying Lin, Zilun Shao, Yang Chang, Hexuan Wang, Yi Wu, Tao Zhang, Yu Du

**Affiliations:** 1 Department of Epidemiology and Health Statistics, West China School of Public Health and West China Fourth Hospital, Sichuan University, Chengdu, Sichuan People’s Republic of China; 2 Health Emergency Management Research Center, West China-PUMC C.C. Chen Institute of Health, Sichuan University, Chengdu, Sichuan People’s Republic of China; 3 Department of Neurosurgery, West China Hospital of Sichuan University, Chengdu, Sichuan People’s Republic of China; 4 Department of Emergency and Critical Care Medicine, West China School of Public Health, West China Fourth Hospital, Sichuan University, Chengdu, Sichuan People’s Republic of China

**Keywords:** Mortality outcome prediction, Intracerebral hemorrhage, Machine learning, Ensemble learning, Outlier detection, Imbalanced data

## Abstract

**Background:**

Outliers and class imbalance in medical data could affect the accuracy of machine learning models. For physicians who want to apply predictive models, how to use the data at hand to build a model and what model to choose are very thorny problems. Therefore, it is necessary to consider outliers, imbalanced data, model selection, and parameter tuning when modeling.

**Methods:**

This study used a joint modeling strategy consisting of: outlier detection and removal, data balancing, model fitting and prediction, performance evaluation. We collected medical record data for all ICH patients with admissions in 2017–2019 from Sichuan Province. Clinical and radiological variables were used to construct models to predict mortality outcomes 90 days after discharge. We used stacking ensemble learning to combine logistic regression (LR), random forest (RF), artificial neural network (ANN), support vector machine (SVM), and k-nearest neighbors (KNN) models. Accuracy, sensitivity, specificity, AUC, precision, and F1 score were used to evaluate model performance. Finally, we compared all 84 combinations of the joint modeling strategy, including training set with and without cross-validated committees filter (CVCF), five resampling techniques (random under-sampling (RUS), random over-sampling (ROS), adaptive synthetic sampling (ADASYN), Borderline synthetic minority oversampling technique (Borderline SMOTE), synthetic minority oversampling technique and edited nearest neighbor (SMOTEENN)) and no resampling, seven models (LR, RF, ANN, SVM, KNN, Stacking, AdaBoost).

**Results:**

Among 4207 patients with ICH, 2909 (69.15%) survived 90 days after discharge, and 1298 (30.85%) died within 90 days after discharge. The performance of all models improved with removing outliers by CVCF except sensitivity. For data balancing processing, the performance of training set without resampling was better than that of training set with resampling in terms of accuracy, specificity, and precision. And the AUC of ROS was the best. For seven models, the average accuracy, specificity, AUC, and precision of RF were the highest. Stacking performed best in F1 score. Among all 84 combinations of joint modeling strategy, eight combinations performed best in terms of accuracy (0.816). For sensitivity, the best performance was SMOTEENN + Stacking (0.662). For specificity, the best performance was CVCF + KNN (0.987). Stacking and AdaBoost had the best performances in AUC (0.756) and F1 score (0.602), respectively. For precision, the best performance was CVCF + SVM (0.938).

**Conclusion:**

This study proposed a joint modeling strategy including outlier detection and removal, data balancing, model fitting and prediction, performance evaluation, in order to provide a reference for physicians and researchers who want to build their own models. This study illustrated the importance of outlier detection and removal for machine learning and showed that ensemble learning might be a good modeling strategy. Due to the low imbalanced ratio (IR, the ratio of majority class and minority class) in this study, we did not find any improvement in models with resampling in terms of accuracy, specificity, and precision, while ROS performed best on AUC.

**Supplementary Information:**

The online version contains supplementary material available at 10.1186/s12911-022-02018-x.

## Background

With the development of information technology, medical data is becoming huge. Many researchers analyze electronic medical records data to provide reference for medical diagnosis, treatment, and prognosis. And machine learning methods have been widely used in medical field. However, medical data may suffer from outliers and class imbalance, which could affect the performance of machine learning models [1, 2]. Therefore, it is necessary to effectively process outliers and imbalanced data in modeling to improve the accuracy of model prediction.

Outlier detection is the process of finding observations that are far from most of the observations. Many studies have shown that removing outliers will improve classification accuracy [3–7]. Podgorelec et al. and Li et al. used outlier detection techniques to remove the detected outliers from training set, and improved the classification accuracy of machine learning methods [5, 7]. There is a lot of outlier detection techniques, and there is no consensus on which method should be used. The cross-validated committees filter (CVCF) [8] is an ensemble filter based on majority voting. CVCF has no complicated parameter settings, and does not need to set threshold for dividing outliers and inliers [8, 9]. Therefore, this study adopts CVCF as an example for outlier detection and removal in modeling.

The performance of machine learning can be affected by class imbalance [1]. In general, the performance of classifier decreases with the increase of imbalanced ratio (IR, the ratio of majority class and minority class). However, IR is not the only factor affecting the performance of classifiers. Class overlapping is also responsible for the decrease in performance of classifiers [10]. Although the IR is not very high, the performance of the classifier can significantly decrease when the classes are highly overlapped. A hybrid resampling method called synthetic minority oversampling technique and edited nearest neighbor (SMOTEENN) [11] was proposed not only to balance the training set but also to remove noisy examples lying on the wrong side of the decision border, which might be caused by SMOTE [11]. And, some studies also showed that the model performance after hybrid resampling was better than that of single resampling [11, 12]. Therefore, several commonly used resampling methods, such as random under-sampling (RUS), random over-sampling (ROS), adaptive synthetic sampling (ADASYN) [13], Borderline SMOTE [14], and SMOTEENN, are used to balance the training set.

Machine learning methods can discover non-linear relationships and explore deeper information in data, and they have great potential for prediction. Although machine learning methods are widely used, the performance of machine learning methods will vary from one data to another, and no one method can always perform well for all data. For example, in the field of intracerebral hemorrhage (ICH) mortality and prognosis prediction. Guo et al. used logistic regression (LR), random forest (RF), support vector machine (SVM), and other methods to predict 90-day functional outcome of patients with ICH, and LR had the highest AUC of 0.89 [15]. Bacchi et al. used four methods, including LR, RF, decision trees (DT), and artificial neural network (ANN), to predict in-hospital mortality of patients with stroke, and LR performed the best with an AUC of 0.90 [16]. Nie et al. used nearest neighbors, DT, ANN, AdaBoost, RF to predict in-hospital mortality of patients with cerebral hemorrhage in intensive care units, and RF had the highest AUC of 0.819 [17]. The other four studies also achieved good performance (high AUC) using RF [18–21]. Lim et al. used SVM to predict 30-day mortality and 90-day poor functional outcome of ICH patients with good AUC performance of 0.9 and 0.883, respectively [22].

Stacking ensemble learning [23] which combines different single classifiers usually performs better than a single classifier [24]. And, it has been increasingly used in medicine in recent years and achieved good performance, for example, predicting the prognosis of patients with glioma [25], predicting adult outcomes in childhood-onset ADHD [26], predicting the recurrence of colorectal cancer [27]. Therefore, we use stacking ensemble learning to combine different machine learning methods which were applied in the prognosis and mortality prediction of patients with ICH.

In this study, we propose a joint modeling strategy to provide reference for physicians and researchers who want to build their own models. It consists of outlier detection and removal, data balancing, model fitting and prediction, performance evaluation.

## Materials and methods

### Data sources

This is a retrospective study, and the data was extracted from the database of Comprehensive Data Collection and Decision Support System for health statistics in Sichuan Province (CDCDS). This database was built by the Sichuan government on January 1, 2017 and covers all ICH admissions in the province. It includes the information of medical records from all general hospitals and community hospitals in Sichuan. We collected medical records information for all ICH patients with admissions in 2017–2019. Patients were identified by International Classification of Diseases, Tenth Revision, Clinical Modification (ICD-10-CM). The patients with nontraumatic intracerebral hemorrhage (I61) were considered in the study.

Medical record information includes clinical and radiological information of the patient at the time of hospitalization. Clinical variables included age, gender, Glasgow Coma Scale (GCS) score at admission, the presence of chronic comorbidity (hypertension and diabetes), treatment (surgery or not), and infection or not. GCS score at admission was estimated and determined by physicians. Hypertension and diabetes are either diagnosed by doctors or self-reported by patients. Treatment refers to whether or not patients had surgery while in the hospital. Infection refers to whether patients developed infection after surgery.

Radiological variables were determined by clinicians using head computed tomography (CT) scans, including ICH location (supratentorial superficial, supratentorial deep, cerebellar, brain stem, intraventricular hemorrhage (IVH)), hematoma volume (measured by the ABC/2 method). ICH location and hematoma volume were estimated and determined by physicians. These variables were regularly collected during hospitalization of patients with ICH.

The outcome of this study was whether patients died within 90 days after discharge. The 90-day mortality was from Ministry of Civil Affairs through unique personal identification numbers.

### Variable selection

We divided age into five categories (40–54, 55–64, 65–74, 75–84, ≥ 85 years). According to clinical criteria, GCS score at admission was divided into three categories (13–15, 9–12, 3–8), indicating mild coma, moderate coma, and severe coma respectively.

In this study, the data has only 10 independent variables, which are not high-dimensional data, so univariate analysis was used to select variables. Because the data are all categorical variables, the chi-square test or Fisher exact test was used to select variables.

The results of univariate analysis showed that age and diabetes have no statistical significance. Considering that the *P* value of age was close to 0.05 and age was an important factor for ICH, the age variable was used for modeling in this study. Therefore, in addition to diabetes, 9 predictors were used for modeling, including age, gender, GCS score at admission, hypertension, surgery, infection, ICH location, supratentorial hemorrhage volume, and infratentorial hemorrhage volume.

### Joint modeling strategy

**Fig. 1:**
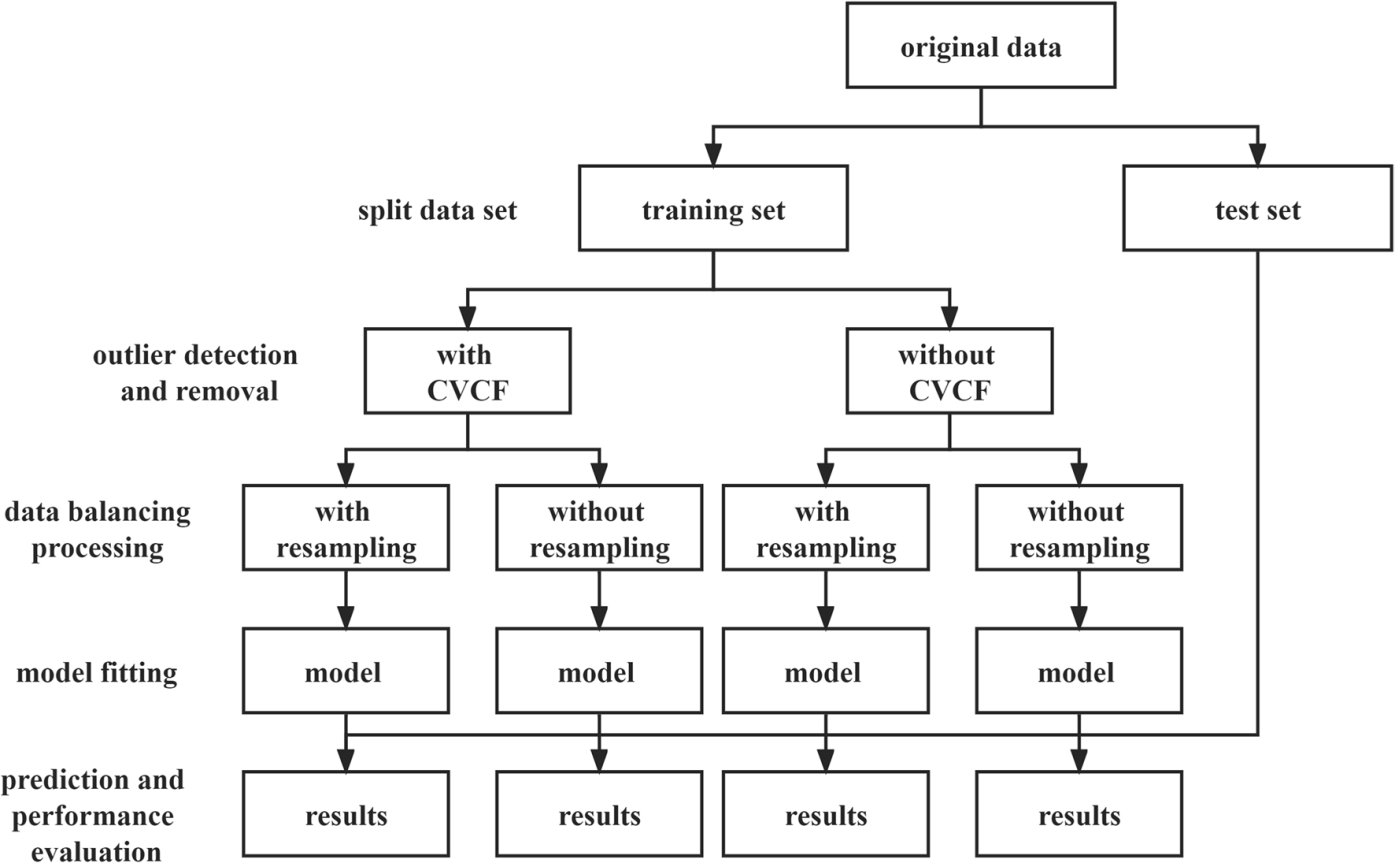
The joint modeling strategy flowchart

Physicians can use information of patients with ICH at the time of hospitalization to predict 90-day mortality after discharge. After ICH patients are admitted to the hospital and treated (after relevant variables were collected), the physicians could give advice to patients (whether to continue treatment or not) based on clinical experience and a prediction of model. However, for physicians and researchers, there are many factors that need to be considered in modeling, such as outliers, imbalanced data, model selection, and parameter tuning. This study shows the use of different methods for handling outliers, imbalanced data and model selection. This joint modeling strategy includes the following steps: outlier detection and removal, data balancing, model fitting and prediction, performance evaluation. To emphasize the importance of outlier removal and data balancing processing, we compared the model performance with and without the corresponding processing. The flow chart is shown in Fig. 1.

We used 10-fold cross-validation (CV) to estimate the results of models, and the IR of each fold remained the same. The final results were the average of the results of 10 test sets. The 95% confidence interval (95% *CI*) of the results were estimated from the results of the 10 test sets.

#### Step 1 outlier detection and removal

In this study, we used CVCF to detect and remove outliers. The R 4.0.2 and “NoiseFiltersR” library were used to implement the CVCF. The parameters of CVCF were set to the default settings in R. We removed outliers detected by CVCF from the training set before further analysis.

In this study, missing values were not processed because there were no missing values.

#### Step 2 data balancing

Although the IR of this study is not very high, we still want to provide physicians with reference for imbalanced data processing methods.

Five resampling methods, including random under-sampling (RUS), random over-sampling (ROS), adaptive synthetic sampling (ADASYN), Borderline SMOTE, SMOTEENN, were used to balance the training set according to outcome variable. The python 3.8.3 and scikit-learn library were used to implement resampling methods. The parameters of resampling methods were set to the default settings in python.

#### Step 3 model fitting and prediction

Stacking ensemble learning was used to combine different machine learning methods which were applied in the prediction of patients with ICH.

**Fig. 2:**
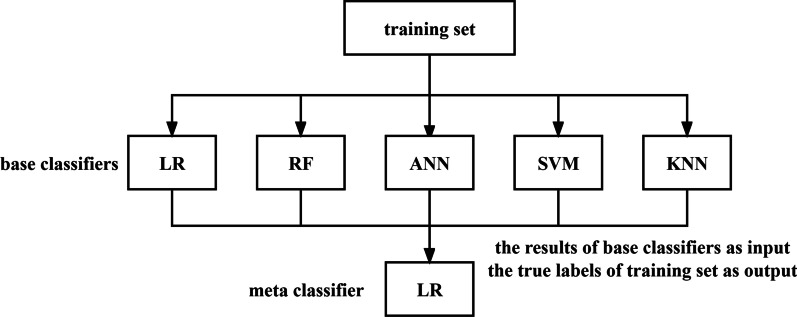
Stacking model

It consists of a two-stage modeling process. In the first stage, different methods (base classifiers) are built on the training set. In the second stage, the meta classifier is trained with the results of the base classifiers as input and the true labels of training set as output. In this study, logistic regression (LR), random forest (RF), artificial neural network (ANN), support vector machine (SVM), and k-nearest neighbors (KNN), which were commonly used, were used as the base classifiers. There is no general criterion for the selection of the meta classifier. Therefore, LR, the classical method, was chosen as the meta classifier. The stacking model is shown in Fig. 2.

Ensemble learning generally includes bagging, boosting, and stacking. Therefore, we also compared three ensemble learning methods. For bagging, random forest (RF) was chosen because it is commonly used and robust [28]. For boosting, we chose the most famous and classic methods called AdaBoost [29]. All combinations of the joint modeling strategy are shown in Table 1. The optimal parameters for each model were selected by grid search using 5-fold cross validation, and the parameter settings were shown in Table 2.

**Table 1:** All combinations of joint modeling strategy

Step	Method	Number
Outlier detection and removal	Without CVCF, With CVCF	2
Data balancing processing	Original, RUS, ROS, ADASYN, Borderline SMOTE, SMOTEENN	6
Models	LR, RF, ANN, SVM, KNN, Stacking, AdaBoost	7
Total	–	84

**Table 2:** The parameter settings

Models	Packages	Parameters to be tuned	Parameters ranges	Optimal parameters
LR	–	–	–	–
RF	randomForest	mtry: number of randomly selected variables	mtry = 1:9	mtry = 5
ANN	nnet	size: numbers of hidden units, decay: weight decay	Size = 1:9, Decay = (0, 0.1, 0.01, 5e-4)	Size = 5, Decay = 0.01
SVM	Kernlab	sigma: Sigma* , C: cost	Kernel = Radial basis function Kernel, C = (0.25, 0.50, 1) **	C = 1
KNN	–	k: number of neighbors	k = (5, 7, 9) **	k = 5
Stacking	caretEnsemble	–	–	–
AdaBoost	fastAdaboost	nIter: number of trees	nIter=(10,20,50,100,150,200,300,500)	nIter = 20

–: No parameter needed to be tunned; *: The optimal value was automatically tuned by R software; **: The parameters ranges were automatically selected by R software

#### Step 4 performance evaluation

We used the confusion matrix for the performance evaluations [30]. Confusion matrix represents counts from predicted and actual values. In this study, six indicators were selected to evaluate model performance, namely accuracy, sensitivity (recall), specificity, precision (Positive Predictive Value, PPV), F1 score, the area under the receiver operating characteristics curve (AUC). We chose 0.5 as the threshold to obtain all these metrics. A larger value for all these six indicators indicates better model performance.

All analyses were performed using R 4.0.2 and Python 3.8.3.

## Results

### Descriptive analysis and variable selection

A total of 4207 patients with ICH were considered in this study. The baseline characteristics for all patients are presented in Table 3. Among 4207 patients, 2909 (69.15%) survived 90 days after discharge and 1298 (30.85%) died within 90 days after discharge. In the univariate analyses, age group and diabetes were not statistically significant. Considering that 99.76% of the patients in this study did not have diabetes, and diabetes was not statistically significant, diabetes was not included in the prediction models in this study.

**Table 3:** Patient baseline characteristics

	Death (*n* = 1298) (%)	Survival (*n* = 2909) (%)	${\chi }^{2}$	P*
Age			9.10	0.059
40–54	308 (23.7)	591 (20.3)		
55–64	294 (22.7)	648 (22.3)		
65–74	434 (33.4)	1032 (35.5)		
75–84	235 (18.1)	551 (18.9)		
≥ 85	27 (2.1)	87 (3.0)		
Gender			28.92	< 0.001
Male	788 (60.7)	1506 (51.8)		
Female	510 (39.3)	1403 (48.2)		
GCS			23.51	< 0.001
13–15	1158 (89.2)	2706 (93.0)		
9–12	107 (8.3)	175 (6.0)		
3–8	33 (2.5)	28 (1.0)		
Hypertension			13.98	< 0.001
No	977 (75.3)	2338 (80.4)		
Yes	321 (24.7)	571 (19.6)		
Diabetes			0.08	0.509
No	1294 (99.7)	2903 (99.8)		
Yes	4 (0.3)	6 (0.2)		
Surgery			148.11	< 0.001
No	1057 (81.4)	2725 (93.7)		
Yes	241 (18.6)	184 (6.3)		
Infection			786.05	< 0.001
No	811 (62.5)	2780 (95.6)		
Yes	487 (37.5)	129 (4.4)		
ICH location			168.50	< 0.001
Supratentorial superficial	1001 (77.1)	2480 (85.3)		
Supratentorial deep	132 (10.2)	239 (8.2)		
Cerebellar	81 (6.2)	157 (5.4)		
Brain stem	82 (6.3)	9 (0.3)		
IVH	2 (0.1)	24 (0.8)		
Supratentorial hemorrhage volume			185.46	< 0.001
< 30ml	1040 (80.1)	2733 (93.9)		
≥ 30ml	258 (19.9)	176 (6.1)		
Infratentorial hemorrhage volume			6.15	0.013
< 10ml	1285 (99.0)	2898 (99.6)		
≥ 10ml	13 (1.0)	11 (0.4)		

*: The *P* value of diabetes was calculated by Fisher exact test; The *P* values of the remaining variables were calculated by chi-square test

### Comparison of training set with and without CVCF

**Fig. 3:**
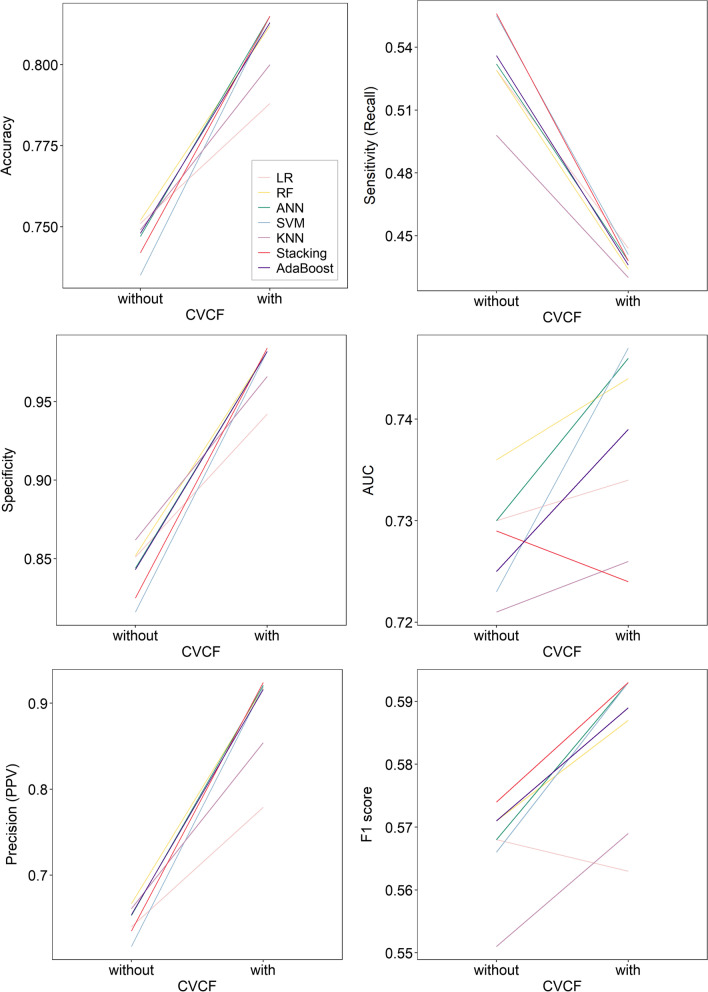
The average performance of 7 models on training set with and without CVCF

Figure 3 shows the average performance of LR, RF, ANN, SVM, KNN, Stacking and AdaBoost on training set with and without CVCF. As can be seen from the figure, with CVCF, the accuracy, specificity, and precision of all models were improved, but the sensitivity was the opposite. The AUC of training set with CVCF were better than that of training set without CVCF, except for stacking model. Similarly, the F1 score of all models except LR improved with CVCF. Overall, removing the detected outliers from training set could improve the performance of some machine learning models.

### Comparison of training set with and without resampling

We calculated the performance of 7 models under each resampling method and ranked from largest to smallest. The smaller the rank is, the better the resampling method performs under the data of this study. Table 4 shows the average performance of 7 models under each resampling method. Table 5 shows the rank of the average performance of each resampling method.

As illustrated in Tables 4 and 5, the accuracy, specificity, and precision of the training set without resampling were better than that of the training set with resampling, but the sensitivity was the opposite. Among the five resampling methods, SMOTEENN showed the greatest increase in sensitivity. The resampling methods can improve the sensitivity of models, but at the cost of reducing the specificity. For AUC and F1 score, different models performed differently under different resampling methods. Combining the performance of each model, the AUC of training set with ROS was the highest. The F1 score of training set with RUS was the highest, followed by ROS. Taking all indicators into account, training set with RUS performed the best, followed by training set with ROS and training set without resampling.

**Table 4:** The average performance of 7 models under each resampling method

	Resampling	Models							Average
		LR	RF	ANN	SVM	KNN	Stacking	AdaBoost	
Acc*	Original	0.792	0.812	0.812	0.811	0.797	0.815	0.815	**0.808**
	RUS	0.786	0.801	0.799	0.790	0.795	0.794	0.800	0.795
	ROS	0.784	0.796	0.796	0.781	0.780	0.790	0.794	0.789
	ADASYN	0.768	0.785	0.777	0.769	0.777	0.781	0.782	0.777
	BSMOTE*	0.751	0.766	0.767	0.758	0.756	0.762	0.763	0.760
	SMOTEENN	0.738	0.732	0.733	0.738	0.744	0.727	0.730	0.735
	Average	0.770	**0.782**	0.781	0.774	0.775	0.778	0.781	0.777
Sen*	Original	0.408	0.425	0.426	0.428	0.382	0.436	0.443	0.421
	RUS	0.493	0.466	0.471	0.492	0.469	0.484	0.464	0.477
	ROS	0.499	0.477	0.489	0.511	0.452	0.499	0.479	0.487
	ADASYN	0.470	0.480	0.484	0.500	0.480	0.492	0.480	0.484
	BSMOTE*	0.514	0.501	0.502	0.511	0.496	0.520	0.504	0.507
	SMOTEENN	0.534	0.540	0.541	0.545	0.504	0.553	0.545	**0.537**
	Average	0.486	0.482	0.486	**0.498**	0.464	0.497	0.486	0.486
Spe*	Original	0.962	0.984	0.985	0.982	0.982	0.984	0.981	**0.980**
	RUS	0.916	0.952	0.947	0.924	0.942	0.933	0.950	0.938
	ROS	0.911	0.940	0.933	0.901	0.927	0.920	0.935	0.924
	ADASYN	0.902	0.921	0.908	0.889	0.909	0.911	0.917	0.908
	BSMOTE*	0.857	0.885	0.887	0.869	0.871	0.871	0.879	0.874
	SMOTEENN	0.829	0.819	0.819	0.826	0.851	0.805	0.812	0.823
	Average	0.896	**0.917**	0.913	0.898	0.914	0.904	0.912	0.908
AUC	Original	0.728	0.740	0.748	0.736	0.730	0.740	0.745	0.738
	RUS	0.738	0.749	0.740	0.737	0.734	0.734	0.736	0.738
	ROS	0.736	0.751	0.750	0.744	0.734	0.747	0.743	**0.744**
	ADASYN	0.729	0.738	0.732	0.732	0.715	0.716	0.719	0.726
	BSMOTE*	0.734	0.740	0.733	0.734	0.720	0.730	0.730	0.732
	SMOTEENN	0.726	0.721	0.724	0.727	0.705	0.692	0.719	0.716
	Average	0.732	**0.740**	0.738	0.735	0.723	0.726	0.732	0.732
Pre*	Original	0.835	0.927	0.928	0.920	0.905	0.925	0.912	**0.907**
	RUS	0.730	0.831	0.821	0.786	0.787	0.801	0.825	0.797
	ROS	0.724	0.798	0.800	0.743	0.738	0.776	0.789	0.767
	ADASYN	0.691	0.773	0.755	0.737	0.733	0.764	0.765	0.745
	BSMOTE*	0.638	0.736	0.733	0.724	0.701	0.728	0.729	0.713
	SMOTEENN	0.640	0.691	0.686	0.698	0.681	0.684	0.692	0.682
	Average	0.710	**0.793**	0.787	0.768	0.758	0.780	0.785	0.769
F1*	Original	0.546	0.581	0.582	0.582	0.535	0.591	0.595	0.573
	RUS	0.585	0.590	0.591	0.591	0.585	0.591	0.587	**0.589**
	ROS	0.587	0.591	0.596	0.590	0.558	0.593	0.589	0.586
	ADASYN	0.554	0.579	0.574	0.574	0.571	0.582	0.577	0.573
	BSMOTE*	0.558	0.573	0.575	0.571	0.558	0.577	0.571	0.569
	SMOTEENN	0.560	0.561	0.563	0.570	0.553	0.564	0.563	0.562
	Average	0.565	0.579	0.580	0.580	0.560	**0.583**	0.580	0.575

*BSMOTE: Borderline SMOTE; Acc: Accuracy; Sen: Sensitivity; Spe: Specificity; Pre: Precision; F1: F1 score; Bold indicates the best value

**Table 5:** The rank of the average performance of each resampling method

	Accuracy	Sensitivity	Specificity	AUC	Precision	F1 score	Sum*	Rank*
Original	1	6	1	2.5	1	3.5	15	2.5
RUS	2	5	2	2.5	2	1	14.5	1
ROS	3	3	3	1	3	2	15	2.5
ADASYN	4	4	4	5	4	3.5	24.5	4
BSMOTE*	5	2	5	4	5	5	26	5
SMOTEENN	6	1	6	6	6	6	31	6

*BSMOTE: Borderline SMOTE; Lower rank is better; Sum = sum of ranks of six indicators; Rank = rank of sum of ranks

### Comparison of 7 models

Table 4 shows the performance of each model under different resampling methods. Table 6 shows the rank of the average performance of each model.

As illustrated in Tables 4 and 6, different models performed differently on different resampling methods. The average accuracy, specificity, AUC and precision of RF were the highest, indicating that RF performed best in distinguishing between patient survival and death. Stacking had good performance in the two indicators of F1 score (ranked 1st), and sensitivity (ranked 2nd). Taking all indicators into account, RF performed best, followed by ANN, AdaBoost and stacking. Compared with LR, SVM, KNN, the performances of ensemble learning were better. For physicians who do not know what model to choose, ensemble learning may be a good choice.

**Table 6:** The rank of the average performance of each model

	Accuracy	Sensitivity	Specificity	AUC	Precision	F1 score	Sum*	Rank*
LR	7	4	7	4.5	7	6	35.5	7
RF	1	6	1	1	1	5	15	1
ANN	2.5	4	3	2	2	3	16.5	2
SVM	6	1	6	3	5	3	24	5
KNN	5	7	2	7	6	7	34	6
Stacking	4	2	5	6	4	1	22	4
AdaBoost	2.5	4	4	4.5	3	3	21	3

*Lower rank is better; Sum = sum of ranks of six indicators; Rank = rank of sum of ranks

### Comparison of all 84 combinations of the joint modeling strategy

Table 7 shows the performance of all 84 combinations of joint modeling strategy. The performance with 95% *CI* of all 84 combinations of joint modeling strategy is shown in Additional file 1. There were eight combinations that performed the best in terms of accuracy (0.816), namely AdaBoost, CVCF + ANN, CVCF + SVM, CVCF + Stacking, CVCF + RUS + Stacking, CVCF + BSMOTE + SVM, CVCF + SMOTEENN + SVM, and CVCF + SMOTEENN + AdaBoost. For sensitivity, the best performance was SMOTEENN + Stacking (0.662). For specificity, the best performance was CVCF + KNN (0.987). For AUC, the best performance was Stacking (0.756). For precision, the best performance was CVCF + SVM (0.938). For F1 score, the best performance was AdaBoost (0.602). Taken together, the joint modeling strategy of CVCF and ensemble learning performed better.

**Table 7:** The performance of all 84 combinations of joint modeling strategy

CVCF	Resampling	Models	Accuracy	Sensitivity	Specificity	AUC	Precision	F1
No	Original	LR	0.787	0.424	0.948	0.733	0.784	0.550
No	Original	RF	0.811	0.426	0.983	0.743	0.920	0.581
No	Original	ANN	0.809	0.415	0.985	0.751	0.925	0.572
No	Original	SVM	0.807	0.420	0.979	0.725	0.903	0.571
No	Original	KNN	0.798	0.395	0.977	0.737	0.886	0.545
No	Original	Stacking	0.813	0.435	0.981	**0.756**	0.916	0.588
No	Original	AdaBoost	**0.816**	0.455	0.977	0.743	0.897	**0.602**
No	RUS	LR	0.777	0.518	0.893	0.738	0.684	0.589
No	RUS	RF	0.789	0.498	0.919	0.750	0.736	0.592
No	RUS	ANN	0.784	0.504	0.909	0.739	0.714	0.590
No	RUS	SVM	0.766	0.547	0.864	0.728	0.644	0.591
No	RUS	KNN	0.785	0.485	0.920	0.744	0.730	0.581
No	RUS	Stacking	0.773	0.533	0.881	0.745	0.669	0.591
No	RUS	AdaBoost	0.786	0.496	0.915	0.738	0.727	0.587
No	ROS	LR	0.774	0.527	0.884	0.736	0.671	0.589
No	ROS	RF	0.784	0.513	0.906	0.750	0.708	0.594
No	ROS	ANN	0.778	0.537	0.886	0.750	0.681	0.599
No	ROS	SVM	0.751	0.572	0.830	0.740	0.603	0.586
No	ROS	KNN	0.775	0.458	0.917	0.733	0.713	0.555
No	ROS	Stacking	0.767	0.557	0.860	0.752	0.642	0.596
No	ROS	AdaBoost	0.778	0.514	0.897	0.740	0.690	0.588
No	ADASYN	LR	0.761	0.516	0.870	0.722	0.640	0.570
No	ADASYN	RF	0.757	0.523	0.862	0.737	0.629	0.569
No	ADASYN	ANN	0.740	0.531	0.834	0.711	0.591	0.557
No	ADASYN	SVM	0.726	0.564	0.798	0.718	0.556	0.558
No	ADASYN	KNN	0.748	0.514	0.853	0.706	0.610	0.556
No	ADASYN	Stacking	0.749	0.548	0.839	0.718	0.605	0.574
No	ADASYN	AdaBoost	0.751	0.523	0.853	0.706	0.615	0.564
No	BSMOTE*	LR	0.729	0.584	0.794	0.732	0.562	0.571
No	BSMOTE	RF	0.720	0.570	0.788	0.737	0.549	0.558
No	BSMOTE	ANN	0.720	0.564	0.791	0.724	0.548	0.555
No	BSMOTE	SVM	0.701	0.584	0.755	0.720	0.520	0.547
No	BSMOTE	KNN	0.707	0.563	0.771	0.718	0.529	0.543
No	BSMOTE	Stacking	0.710	0.602	0.758	0.733	0.529	0.561
No	BSMOTE	AdaBoost	0.713	0.572	0.776	0.721	0.538	0.552
No	SMOTEENN	LR	0.680	0.603	0.715	0.718	0.497	0.539
No	SMOTEENN	RF	0.651	0.646	0.654	0.696	0.459	0.534
No	SMOTEENN	ANN	0.652	0.642	0.656	0.706	0.459	0.533
No	SMOTEENN	SVM	0.661	0.645	0.669	0.707	0.474	0.542
No	SMOTEENN	KNN	0.683	0.570	0.735	0.686	0.501	0.526
No	SMOTEENN	Stacking	0.638	**0.662**	0.628	0.672	0.449	0.531
No	SMOTEENN	AdaBoost	0.645	0.654	0.642	0.703	0.460	0.535
Yes	Original	LR	0.797	0.393	0.977	0.722	0.885	0.543
Yes	Original	RF	0.812	0.424	0.986	0.737	0.933	0.581
Yes	Original	ANN	**0.816**	0.436	0.985	0.745	0.931	0.592
Yes	Original	SVM	**0.816**	0.436	0.986	0.746	**0.938**	0.593
Yes	Original	KNN	0.796	0.368	**0.987**	0.724	0.924	0.525
Yes	Original	Stacking	**0.816**	0.436	0.986	0.725	0.933	0.593
Yes	Original	AdaBoost	0.814	0.431	0.985	0.747	0.927	0.587
Yes	RUS	LR	0.794	0.468	0.939	0.738	0.775	0.582
Yes	RUS	RF	0.814	0.433	0.984	0.748	0.925	0.588
Yes	RUS	ANN	0.815	0.437	0.984	0.742	0.928	0.593
Yes	RUS	SVM	0.815	0.437	0.984	0.746	0.928	0.592
Yes	RUS	KNN	0.805	0.453	0.963	0.725	0.845	0.589
Yes	RUS	Stacking	**0.816**	0.435	0.986	0.724	0.933	0.592
Yes	RUS	AdaBoost	0.814	0.433	0.984	0.734	0.923	0.588
Yes	ROS	LR	0.795	0.471	0.939	0.735	0.777	0.585
Yes	ROS	RF	0.809	0.440	0.974	0.752	0.889	0.587
Yes	ROS	ANN	0.814	0.440	0.981	0.749	0.918	0.593
Yes	ROS	SVM	0.811	0.451	0.972	0.748	0.883	0.594
Yes	ROS	KNN	0.786	0.445	0.938	0.734	0.764	0.561
Yes	ROS	Stacking	0.813	0.440	0.980	0.742	0.911	0.591
Yes	ROS	AdaBoost	0.810	0.443	0.974	0.746	0.888	0.589
Yes	ADASYN	LR	0.776	0.424	0.934	0.736	0.741	0.538
Yes	ADASYN	RF	0.813	0.437	0.981	0.738	0.917	0.590
Yes	ADASYN	ANN	0.814	0.437	0.982	0.752	0.919	0.590
Yes	ADASYN	SVM	0.813	0.437	0.981	0.746	0.917	0.590
Yes	ADASYN	KNN	0.806	0.446	0.966	0.724	0.856	0.585
Yes	ADASYN	Stacking	0.814	0.436	0.983	0.714	0.923	0.590
Yes	ADASYN	AdaBoost	0.813	0.436	0.981	0.732	0.915	0.589
Yes	BSMOTE	LR	0.773	0.445	0.919	0.736	0.714	0.546
Yes	BSMOTE	RF	0.813	0.433	0.983	0.743	0.922	0.588
Yes	BSMOTE	ANN	0.815	0.441	0.982	0.742	0.918	0.594
Yes	BSMOTE	SVM	**0.816**	0.439	0.984	0.749	0.927	0.594
Yes	BSMOTE	KNN	0.804	0.429	0.971	0.723	0.872	0.574
Yes	BSMOTE	Stacking	0.815	0.438	0.984	0.727	0.926	0.593
Yes	BSMOTE	AdaBoost	0.813	0.436	0.982	0.738	0.919	0.589
Yes	SMOTEENN	LR	0.795	0.466	0.942	0.735	0.783	0.582
Yes	SMOTEENN	RF	0.814	0.435	0.983	0.746	0.922	0.589
Yes	SMOTEENN	ANN	0.814	0.440	0.981	0.743	0.914	0.593
Yes	SMOTEENN	SVM	**0.816**	0.445	0.982	0.747	0.921	0.598
Yes	SMOTEENN	KNN	0.805	0.438	0.968	0.724	0.862	0.580
Yes	SMOTEENN	Stacking	**0.816**	0.444	0.982	0.711	0.920	0.597
Yes	SMOTEENN	AdaBoost	0.815	0.437	0.983	0.735	0.924	0.591

* BSMOTE: Borderline SMOTE; Bold indicates the best value

## Discussion

Taking ICH as an example, this study presented a joint modeling strategy considering outliers, imbalanced data, model selection, parameter tuning, in order to provide a reference for physicians and researchers interested in constructing similar models. The results of this study show that it is necessary to adopt a joint modeling strategy that considers multiple processing and modeling methods, which can improve the performance of models.

The results of this study illustrate that removing the detected outliers from training set could improve the performance of models. Patients of ICH may get worse or even die after discharge for competitive risks, such as recurrence of ICH, thrombus dislodgement, infection. We did not collect information about these competitive risks and therefore there was no way to predict them. Those deaths that were unpredictable with the information we collected were removed from the training set by CVCF, but kept in the test set, as similar situations may still occur in future datasets. Therefore, this may be the reason why the sensitivity of the model of the training set with CVCF decreased compared to the model of the training set without CVCF. In addition, iForest [31] is also a good choice for outlier detection, but requires multiple attempts to select optimal parameters. There were only ten variables in this study, so variable selection was relatively simple. In case of more variables, more complex methods can be considered, such as Least Absolute Shrinkage and Selection Operator (LASSO) [32].

In terms of data balancing processing, due to the low IR in this study, all resampling methods did not improve the model performance compared to no resampling. But our study also compared 5 resampling methods, which could provide some insights. In the case of a large number of minority samples in this study, ROS achieved the best AUC, which was consistent with the findings of Batista et al. Batista et al. [11] showed that SMOTE + Tomek and SMOTE + ENN were more suitable for data sets with a small number of minority instances. For data sets with larger number of minority instances, the ROS could be a good choice because it is less computationally expensive and it could provide competitive results with the more complex methods [11].

For model selection, this study showed that ensemble learning might be a good choice, such as RF, AdaBoost, and Stacking. For stacking, researchers can choose methods commonly used in their fields as base classifiers. The most classic LR was selected as the meta classifier in this study, and researchers can try other more complex methods as meta classifier to obtain better performance.

This study has some strengths. Firstly, the data of this study is large and comes from multi-center population of Sichuan Province. Secondly, stacking was used to combine several common machine learning methods. Finally, the joint modeling strategy considering outliers, imbalanced data, model selection, and parameter tuning was presented to achieve good prediction performance.

Meanwhile, this study inevitably has several limitations. Firstly, this study is a retrospective design with the inherent risk of bias and lack of a validation cohort. Secondly, this study did not have information about early withdrawal of care, which was an important confounder in ICH research.

The results of this study could shed light upon future work in several ways. First of all, external validation is needed to test the generalizability of this model. Besides, more predictive factors could be considered in this model, so as to improve the prediction performance. Finally, the parameters in this model were selected automatically by software using grid searching, which may result in sub-optimal parameters selection. Further work can focus on expanding the range of parameters selection and considering more comprehensive selection of base and meta classifiers, so as to improve the predictive efficiency.

## Conclusion

This study used information of patients with ICH at the time of hospitalization to predict 90-day mortality after discharge. We proposed a joint modeling strategy that takes into account outliers, imbalanced data, model selection, and parameter tuning, in order to provide reference for physicians and researchers. This study illustrated the importance of outlier detection and removal for machine learning and showed that ensemble learning might be a good modeling strategy. Due to the low IR in this study, we did not find obvious improvement of models with resampling methods in terms of accuracy, specificity, and precision. However, our results also validated that ROS performed comparable to more complex methods on AUC in the case of a large number of minority samples.

## Supplementary Information

**Additional file 1.** Additional file showed the performance with 95% *CI* of all 84 combinations of joint modeling strategy.

## Data Availability

The datasets analyzed during the current study are not publicly available due to privacy but are available from the corresponding author on reasonable request.

## Abbreviations

ICH: Intracerebral hemorrhage
IVH: Intraventricular hemorrhage
CVCF: Cross-validated committees filter
SMOTE: Synthetic minority oversampling technique
ENN: Edited nearest neighbor
SMOTEENN: Synthetic minority oversampling technique and edited nearest neighbor
AUC: Areas under curve
ROC: Receiver operating characteristic
LR: Logistic regression
RF: Random forest
ANN: Artificial neural network
SVM: Support vector machine
KNN: k-nearest neighbors
CI: Confidence interval
GCS: Glasgow coma scale

## References

[1] Japkowicz N, Stephen S (2002). The class imbalance problem: a systematic study. Intell Data Anal.

[2] Tallon-Ballesteros AJ, Riquelme JC. Deleting or keeping outliers for classifier training? *6th**World Congress on Nature and Biologically Inspired Computing**(NaBIC)*. Porto, Portuga, 2014; pp. 281-286.

[3] Fitriyani NL, Syafrudin M, Alfian G, Rhee J (2019). Development of disease prediction model based on ensemble learning approach for diabetes and hypertension. Ieee Access.

[4] Ijaz MF, Attique M, Son Y (2020). Data-driven cervical cancer prediction model with outlier detection and over-sampling methods. Sensors.

[5] Li W, Mo W, Zhang X, Squiers JJ, Lu Y, Sellke EW (2015). Outlier detection and removal improves accuracy of machine learning approach to multispectral burn diagnostic imaging. J Biomed Opt..

[6] Meneghetti L, Terzi M, Del Favero S, Susto GA, Cobelli C (2020). Data-driven anomaly recognition for unsupervised model-free fault detection in artificial pancreas. IEEE Trans Control Syst Technol.

[7] Podgorelec V, Hericko M, Rozman I. Improving mining of medical data by outliers prediction. *18th IEEE Symposium on Computer-Based Medical Systems (CBMS'05)*, 2005, pp. 91-96.

[8] Verbaeten S, Van Assche A, Windeatt T, Roli F (2003). Ensemble methods for noise elimination in classification problems. Multiple classifier systems,.

[9] Afanasyev DO, Fedorova EA (2019). On the impact of outlier filtering on the electricity price forecasting accuracy. Appl Energy.

[10] Lin W-J, Chen JJ (2013). Class-imbalanced classifiers for high-dimensional data. Brief Bioinform.

[11] Batista GEAPA, Prati RC, Monard MC (2004). A study of the behavior of several methods for balancing machine learning training data. ACM SIGKDD Explor Newsl.

[12] Seiffert C, Khoshgoftaar TM, Van Hulse J (2009). Hybrid sampling for imbalanced data. Integr Comput Aided Eng.

[13] He H, Bai Y, Garcia EA, Li S. ADASYN: adaptive synthetic sampling approach for imbalanced learning. *2008 IEEE International Joint Conference on Neural Networks (IEEE World Congress on Computational Intelligence)*, 2008, pp. 1322-1328.

[14] Han H, Wang WY, Mao BH, Huang DS, Zhang XP, Huang GB (2005). Borderline-SMOTE: a new over-sampling method in imbalanced data sets learning. Advances in intelligent computing, Pt 1, proceedings. Lecture notes in computer science.

[15] Guo R, Zhang R, Liu R, Liu Y, Li H, Ma L (2022). Machine learning-based approaches for prediction of patients’ functional outcome and mortality after spontaneous intracerebral hemorrhage. J Pers Med.

[16] Bacchi S, Oakden-Rayner L, Menon DK, Jannes J, Kleinig T, Koblar S (2020). Stroke prognostication for discharge planning with machine learning: a derivation study. J Clin Neurosci.

[17] Nie X, Cai Y, Liu J, Liu X, Zhao J, Yang Z (2021). Mortality prediction in cerebral hemorrhage patients using machine learning algorithms in intensive care units. Front Neurol..

[18] Fernandez-Lozano C, Hervella P, Mato-Abad V, Rodriguez-Yanez M, Suarez-Garaboa S, Lopez-Dequidt I (2021). Random forest-based prediction of stroke outcome. Sci Rep.

[19] Trevisi G, Caccavella VM, Scerrati A, Signorelli F, Salamone GG, Orsini K, et al. Machine learning model prediction of 6-month functional outcome in elderly patients with intracerebral hemorrhage. Neurosurgical Review. 2022;45:2857–67. 10.1007/s10143-022-01802-7 PMC9349060 35522333

[20] Wang HL, Hsu WY, Lee MH, Weng HH, Chang SW, Yang JT (2019). Automatic machine-learning-based outcome prediction in patients with primary intracerebral hemorrhage. Front Neurol.

[21] Zhu F, Pan Z, Tang Y, Fu P, Cheng S, Hou W (2021). Machine learning models predict coagulopathy in spontaneous intracerebral hemorrhage patients in ER. CNS Neurosci Ther.

[22] Lim MJR, Quek RHC, Ng KJ, Loh NW, Lwin S, Teo K (2022). Machine learning models prognosticate functional outcomes better than clinical scores in spontaneous intracerebral haemorrhage. J Stroke Cerebrovasc Dis.

[23] Wolpert DH (1992). Stacked generalization. Neural Netw.

[24] Zhou Z-H (2012). Ensemble methods: foundations and algorithms.

[25] Samara KA, Aghbari ZA, Abusafia A (2021). GLIMPSE: a glioblastoma prognostication model using ensemble learning—a surveillance, epidemiology, and end results study. Health Inf Sci Syst.

[26] Luo Y, Alvarez TL, Halperin JM, Li X (2020). Multimodal neuroimaging-based prediction of adult outcomes in childhood-onset ADHD using ensemble learning techniques. Neuroimage Clin.

[27] Chan HC, Chattopadhyay A, Chuang EY, Lu TP (2021). Development of a gene-based prediction model for recurrence of colorectal cancer using an ensemble learning algorithm. Front Oncol.

[28] Breiman L (2001). Random forests. Mach Learn.

[29] Freund Y, Schapire RE (1997). A decision-theoretic generalization of on-line learning and an application to boosting. J Comput Syst Sci.

[30] Kulkarni A, Chong D, Batarseh FA, Batarseh FA, Yang R (2020). 5 - foundations of data imbalance and solutions for a data democracy.

[31] Liu FT, Ting KM, Zhou Z-H (2012). Isolation-based anomaly detection. Acm Trans Knowl Discov Data.

[32] Tibshirani R (1996). Regression shrinkage and selection via the Lasso. J Royal Stat Soc Ser B-Methodol.

