# Supplementary material for: Joint modeling strategy for using electronic medical records data to build machine learning models: an example of intracerebral hemorrhage
Source: BMC Med Inform Decis Mak. 2022 Oct 25;22:278. doi: 10.1186/s12911-022-02018-x (PMC9594939; doi:10.1186/s12911-022-02018-x)
Supplement: Supplementary file 1 — Additional file 1. Additional file showed the performance with 95% CI of all 84 combinations of joint modeling strategy. [file 12911_2022_2018_MOESM1_ESM.pdf]

CVCF	resampling	model	Accuracy	Sensitivity	Specificity	AUC	Precision	Recall	F1
No	Original	LR	0.787 (0.779-0.794)	0.424 (0.399-0.45)	0.948 (0.943-0.953)	0.733 (0.717-0.748)	0.784 (0.764-0.805)	0.424 (0.399-0.45)	0.55 (0.526-0.574)
No	Original	RF	0.811 (0.803-0.82)	0.426 (0.404-0.448)	0.983 (0.977-0.99)	0.743 (0.729-0.757)	0.92 (0.893-0.947)	0.426 (0.404-0.448)	0.581 (0.559-0.603)
No	Original	ANN	0.809 (0.801-0.817)	0.415 (0.391-0.439)	0.985 (0.978-0.991)	0.751 (0.734-0.767)	0.925 (0.9-0.951)	0.415 (0.391-0.439)	0.572 (0.549-0.594)
No	Original	SVM	0.807 (0.799-0.814)	0.42 (0.395-0.445)	0.979 (0.972-0.986)	0.725 (0.708-0.743)	0.903 (0.877-0.929)	0.42 (0.395-0.445)	0.571 (0.55-0.593)
No	Original	KNN	0.798 (0.789-0.807)	0.395 (0.369-0.421)	0.977 (0.972-0.983)	0.737 (0.722-0.753)	0.886 (0.86-0.912)	0.395 (0.369-0.421)	0.545 (0.519-0.572)
No	Original	Stacking	0.813 (0.805-0.821)	0.435 (0.409-0.462)	0.981 (0.974-0.989)	0.756 (0.741-0.77)	0.916 (0.886-0.946)	0.435 (0.409-0.462)	0.588 (0.565-0.612)
No	Original	AdaBoost	0.816 (0.805-0.826)	0.455 (0.428-0.482)	0.977 (0.971-0.982)	0.743 (0.727-0.758)	0.897 (0.877-0.918)	0.455 (0.428-0.482)	0.602 (0.578-0.627)
No	ROS	LR	0.777 (0.764-0.79)	0.518 (0.491-0.544)	0.893 (0.882-0.904)	0.738 (0.721-0.755)	0.684 (0.661-0.707)	0.518 (0.491-0.544)	0.589 (0.565-0.613)
No	ROS	RF	0.789 (0.775-0.802)	0.498 (0.475-0.522)	0.919 (0.902-0.936)	0.75 (0.736-0.763)	0.736 (0.691-0.78)	0.498 (0.475-0.522)	0.592 (0.568-0.617)
No	ROS	ANN	0.784 (0.771-0.797)	0.504 (0.477-0.532)	0.909 (0.896-0.923)	0.739 (0.722-0.757)	0.714 (0.679-0.749)	0.504 (0.477-0.532)	0.59 (0.565-0.615)
No	ROS	SVM	0.766 (0.751-0.782)	0.547 (0.523-0.572)	0.864 (0.848-0.88)	0.728 (0.715-0.741)	0.644 (0.619-0.669)	0.547 (0.523-0.572)	0.591 (0.57-0.613)
No	ROS	KNN	0.785 (0.778-0.793)	0.485 (0.459-0.511)	0.92 (0.911-0.929)	0.744 (0.729-0.759)	0.73 (0.703-0.756)	0.485 (0.459-0.511)	0.581 (0.56-0.602)
No	ROS	Stacking	0.773 (0.762-0.785)	0.533 (0.504-0.562)	0.881 (0.864-0.899)	0.745 (0.727-0.764)	0.669 (0.636-0.703)	0.533 (0.504-0.562)	0.591 (0.57-0.613)
No	ROS	AdaBoost	0.786 (0.778-0.794)	0.496 (0.473-0.518)	0.915 (0.899-0.932)	0.738 (0.722-0.754)	0.727 (0.69-0.765)	0.496 (0.473-0.518)	0.587 (0.572-0.603)
No	RUS	LR	0.774 (0.763-0.785)	0.527 (0.497-0.557)	0.884 (0.873-0.896)	0.736 (0.72-0.753)	0.671 (0.653-0.69)	0.527 (0.497-0.557)	0.589 (0.568-0.611)
No	RUS	RF	0.784 (0.771-0.798)	0.513 (0.481-0.544)	0.906 (0.895-0.916)	0.75 (0.735-0.765)	0.708 (0.683-0.733)	0.513 (0.481-0.544)	0.594 (0.567-0.621)
No	RUS	ANN	0.778 (0.762-0.795)	0.537 (0.505-0.569)	0.886 (0.869-0.903)	0.75 (0.732-0.768)	0.681 (0.647-0.714)	0.537 (0.505-0.569)	0.599 (0.572-0.626)
No	RUS	SVM	0.751 (0.737-0.764)	0.572 (0.549-0.595)	0.83 (0.812-0.849)	0.74 (0.726-0.753)	0.603 (0.577-0.629)	0.572 (0.549-0.595)	0.586 (0.568-0.604)
No	RUS	KNN	0.775 (0.764-0.786)	0.458 (0.425-0.491)	0.917 (0.904-0.929)	0.733 (0.719-0.746)	0.713 (0.681-0.744)	0.458 (0.425-0.491)	0.555 (0.528-0.582)
No	RUS	Stacking	0.767 (0.751-0.783)	0.557 (0.529-0.586)	0.86 (0.845-0.876)	0.752 (0.736-0.768)	0.642 (0.616-0.667)	0.557 (0.529-0.586)	0.596 (0.572-0.619)
No	RUS	AdaBoost	0.778 (0.764-0.793)	0.514 (0.484-0.544)	0.897 (0.883-0.91)	0.74 (0.726-0.754)	0.69 (0.656-0.723)	0.514 (0.484-0.544)	0.588 (0.56-0.616)
No	ADAYSN	LR	0.761 (0.75-0.772)	0.516 (0.484-0.547)	0.87 (0.856-0.884)	0.722 (0.704-0.74)	0.64 (0.625-0.656)	0.516 (0.484-0.547)	0.57 (0.55-0.59)
No	ADAYSN	RF	0.757 (0.741-0.772)	0.523 (0.489-0.556)	0.862 (0.843-0.88)	0.737 (0.718-0.756)	0.629 (0.588-0.669)	0.523 (0.489-0.556)	0.569 (0.54-0.598)
No	ADAYSN	ANN	0.74 (0.724-0.757)	0.531 (0.498-0.563)	0.834 (0.812-0.856)	0.711 (0.694-0.729)	0.591 (0.552-0.63)	0.531 (0.498-0.563)	0.557 (0.53-0.584)
No	ADAYSN	SVM	0.726 (0.713-0.738)	0.564 (0.532-0.595)	0.798 (0.781-0.816)	0.718 (0.698-0.738)	0.556 (0.533-0.578)	0.564 (0.532-0.595)	0.558 (0.538-0.579)
No	ADAYSN	KNN	0.748 (0.736-0.76)	0.514 (0.485-0.543)	0.853 (0.837-0.869)	0.706 (0.691-0.722)	0.61 (0.578-0.642)	0.514 (0.485-0.543)	0.556 (0.534-0.579)
No	ADAYSN	Stacking	0.749 (0.732-0.766)	0.548 (0.52-0.576)	0.839 (0.82-0.858)	0.718 (0.7-0.736)	0.605 (0.572-0.638)	0.548 (0.52-0.576)	0.574 (0.548-0.6)
No	ADAYSN	AdaBoost	0.751 (0.74-0.762)	0.523 (0.491-0.556)	0.853 (0.839-0.868)	0.706 (0.688-0.723)	0.615 (0.585-0.645)	0.523 (0.491-0.556)	0.564 (0.539-0.589)
No	BSMOTE	LR	0.729 (0.711-0.747)	0.584 (0.556-0.613)	0.794 (0.765-0.823)	0.732 (0.716-0.749)	0.562 (0.526-0.599)	0.584 (0.556-0.613)	0.571 (0.549-0.593)
No	BSMOTE	RF	0.72 (0.7-0.741)	0.57 (0.547-0.593)	0.788 (0.759-0.817)	0.737 (0.721-0.753)	0.549 (0.508-0.591)	0.57 (0.547-0.593)	0.558 (0.531-0.585)
No	BSMOTE	ANN	0.72 (0.703-0.738)	0.564 (0.544-0.584)	0.791 (0.767-0.814)	0.724 (0.701-0.747)	0.548 (0.512-0.585)	0.564 (0.544-0.584)	0.555 (0.531-0.579)
No	BSMOTE	SVM	0.701 (0.678-0.725)	0.584 (0.555-0.612)	0.755 (0.717-0.792)	0.72 (0.699-0.741)	0.52 (0.48-0.561)	0.584 (0.555-0.612)	0.547 (0.521-0.574)
No	BSMOTE	KNN	0.707 (0.682-0.731)	0.563 (0.543-0.583)	0.771 (0.737-0.806)	0.718 (0.698-0.738)	0.529 (0.486-0.571)	0.563 (0.543-0.583)	0.543 (0.515-0.571)
No	BSMOTE	Stacking	0.71 (0.692-0.727)	0.602 (0.575-0.628)	0.758 (0.728-0.787)	0.733 (0.712-0.755)	0.529 (0.499-0.56)	0.602 (0.575-0.628)	0.561 (0.543-0.579)
No	BSMOTE	AdaBoost	0.713 (0.689-0.737)	0.572 (0.548-0.596)	0.776 (0.742-0.811)	0.721 (0.699-0.744)	0.538 (0.496-0.581)	0.572 (0.548-0.596)	0.552 (0.524-0.581)
No	SMOTEENN	LR	0.68 (0.646-0.714)	0.603 (0.568-0.637)	0.715 (0.658-0.772)	0.718 (0.698-0.739)	0.497 (0.446-0.549)	0.603 (0.568-0.637)	0.539 (0.512-0.567)
No	SMOTEENN	RF	0.651 (0.625-0.677)	0.646 (0.61-0.682)	0.654 (0.606-0.702)	0.696 (0.671-0.721)	0.459 (0.427-0.492)	0.646 (0.61-0.682)	0.534 (0.514-0.554)
No	SMOTEENN	ANN	0.652 (0.624-0.679)	0.642 (0.607-0.678)	0.656 (0.614-0.699)	0.706 (0.685-0.727)	0.459 (0.426-0.491)	0.642 (0.607-0.678)	0.533 (0.508-0.558)
No	SMOTEENN	SVM	0.661 (0.626-0.695)	0.645 (0.615-0.675)	0.669 (0.61-0.727)	0.707 (0.686-0.727)	0.474 (0.431-0.517)	0.645 (0.615-0.675)	0.542 (0.518-0.565)
No	SMOTEENN	KNN	0.683 (0.652-0.715)	0.57 (0.527-0.612)	0.735 (0.676-0.794)	0.686 (0.663-0.709)	0.501 (0.457-0.544)	0.57 (0.527-0.612)	0.526 (0.504-0.549)
No	SMOTEENN	Stacking	0.638 (0.603-0.673)	0.662 (0.624-0.699)	0.628 (0.569-0.687)	0.672 (0.647-0.696)	0.449 (0.412-0.487)	0.662 (0.624-0.699)	0.531 (0.507-0.556)
No	SMOTEENN	AdaBoost	0.645 (0.605-0.686)	0.654 (0.617-0.692)	0.642 (0.571-0.712)	0.703 (0.68-0.727)	0.46 (0.415-0.506)	0.654 (0.617-0.692)	0.535 (0.509-0.56)
Yes	Original	LR	0.797 (0.788-0.805)	0.393 (0.369-0.417)	0.977 (0.969-0.985)	0.722 (0.707-0.738)	0.885 (0.851-0.919)	0.393 (0.369-0.417)	0.543 (0.518-0.567)
Yes	Original	RF	0.812 (0.803-0.821)	0.424 (0.395-0.452)	0.986 (0.98-0.992)	0.737 (0.722-0.751)	0.933 (0.909-0.957)	0.424 (0.395-0.452)	0.581 (0.555-0.607)
Yes	Original	ANN	0.816 (0.806-0.825)	0.436 (0.409-0.462)	0.985 (0.979-0.992)	0.745 (0.727-0.763)	0.931 (0.905-0.957)	0.436 (0.409-0.462)	0.592 (0.566-0.617)
Yes	Original	SVM	0.816 (0.807-0.826)	0.436 (0.407-0.466)	0.986 (0.978-0.994)	0.746 (0.735-0.758)	0.938 (0.907-0.969)	0.436 (0.407-0.466)	0.593 (0.567-0.62)
Yes	Original	KNN	0.796 (0.786-0.805)	0.368 (0.341-0.395)	0.987 (0.982-0.992)	0.724 (0.713-0.735)	0.924 (0.896-0.951)	0.368 (0.341-0.395)	0.525 (0.495-0.555)
Yes	Original	Stacking	0.816 (0.806-0.826)	0.436 (0.409-0.464)	0.986 (0.979-0.992)	0.725 (0.703-0.747)	0.933 (0.905-0.96)	0.436 (0.409-0.464)	0.593 (0.567-0.619)
Yes	Original	AdaBoost	0.814 (0.805-0.823)	0.431 (0.404-0.458)	0.985 (0.979-0.99)	0.747 (0.734-0.761)	0.927 (0.904-0.951)	0.431 (0.404-0.458)	0.587 (0.562-0.611)
Yes	ROS	LR	0.794 (0.785-0.802)	0.468 (0.439-0.497)	0.939 (0.932-0.946)	0.738 (0.723-0.753)	0.775 (0.755-0.795)	0.468 (0.439-0.497)	0.582 (0.558-0.606)
Yes	ROS	RF	0.814 (0.805-0.822)	0.433 (0.406-0.459)	0.984 (0.978-0.99)	0.748 (0.733-0.763)	0.925 (0.901-0.95)	0.433 (0.406-0.459)	0.588 (0.564-0.611)
Yes	ROS	ANN	0.815 (0.806-0.825)	0.437 (0.411-0.464)	0.984 (0.976-0.992)	0.742 (0.724-0.759)	0.928 (0.896-0.96)	0.437 (0.411-0.464)	0.593 (0.568-0.617)
Yes	ROS	SVM	0.815 (0.806-0.825)	0.437 (0.409-0.465)	0.984 (0.977-0.991)	0.746 (0.736-0.756)	0.928 (0.9-0.956)	0.437 (0.409-0.465)	0.592 (0.567-0.618)
Yes	ROS	KNN	0.805 (0.797-0.814)	0.453 (0.429-0.476)	0.963 (0.958-0.968)	0.725 (0.714-0.735)	0.845 (0.83-0.86)	0.453 (0.429-0.476)	0.589 (0.568-0.609)
Yes	ROS	Stacking	0.816 (0.806-0.825)	0.435 (0.408-0.461)	0.986 (0.979-0.992)	0.724 (0.701-0.747)	0.933 (0.906-0.96)	0.435 (0.408-0.461)	0.592 (0.567-0.616)
Yes	ROS	AdaBoost	0.814 (0.804-0.823)	0.433 (0.405-0.461)	0.984 (0.978-0.989)	0.734 (0.722-0.747)	0.923 (0.9-0.946)	0.433 (0.405-0.461)	0.588 (0.562-0.614)
Yes	RUS	LR	0.795 (0.787-0.802)	0.471 (0.445-0.498)	0.939 (0.931-0.947)	0.735 (0.719-0.75)	0.777 (0.755-0.799)	0.471 (0.445-0.498)	0.585 (0.565-0.605)
Yes	RUS	RF	0.809 (0.801-0.818)	0.44 (0.413-0.467)	0.974 (0.965-0.984)	0.752 (0.738-0.765)	0.889 (0.857-0.92)	0.44 (0.413-0.467)	0.587 (0.564-0.609)
Yes	RUS	ANN	0.814 (0.804-0.824)	0.44 (0.413-0.467)	0.981 (0.972-0.991)	0.749 (0.735-0.764)	0.918 (0.882-0.955)	0.44 (0.413-0.467)	0.593 (0.568-0.618)
Yes	RUS	SVM	0.811 (0.802-0.819)	0.451 (0.421-0.48)	0.972 (0.959-0.984)	0.748 (0.736-0.76)	0.883 (0.842-0.924)	0.451 (0.421-0.48)	0.594 (0.572-0.616)
Yes	RUS	KNN	0.786 (0.776-0.797)	0.445 (0.419-0.471)	0.938 (0.929-0.947)	0.734 (0.719-0.75)	0.764 (0.738-0.79)	0.445 (0.419-0.471)	0.561 (0.536-0.587)
Yes	RUS	Stacking	0.813 (0.804-0.822)	0.44 (0.412-0.467)	0.98 (0.971-0.989)	0.742 (0.724-0.759)	0.911 (0.878-0.944)	0.44 (0.412-0.467)	0.591 (0.567-0.614)
Yes	RUS	AdaBoost	0.81 (0.8-0.82)	0.443 (0.415-0.47)	0.974 (0.965-0.983)	0.746 (0.728-0.763)	0.888 (0.858-0.918)	0.443 (0.415-0.47)	0.589 (0.564-0.613)
Yes	ADAYSN	LR	0.776 (0.765-0.787)	0.424 (0.394-0.454)	0.934 (0.926-0.941)	0.736 (0.719-0.753)	0.741 (0.72-0.761)	0.424 (0.394-0.454)	0.538 (0.512-0.564)
Yes	ADAYSN	RF	0.813 (0.804-0.823)	0.437 (0.41-0.465)	0.981 (0.974-0.989)	0.738 (0.724-0.753)	0.917 (0.888-0.945)	0.437 (0.41-0.465)	0.59 (0.566-0.614)
Yes	ADAYSN	ANN	0.814 (0.803-0.824)	0.437 (0.408-0.465)	0.982 (0.974-0.99)	0.752 (0.735-0.768)	0.919 (0.888-0.95)	0.437 (0.408-0.465)	0.59 (0.564-0.616)
Yes	ADAYSN	SVM	0.813 (0.804-0.823)	0.437 (0.409-0.465)	0.981 (0.973-0.99)	0.746 (0.734-0.759)	0.917 (0.885-0.95)	0.437 (0.409-0.465)	0.59 (0.565-0.615)
Yes	ADAYSN	KNN	0.806 (0.795-0.816)	0.446 (0.422-0.47)	0.966 (0.958-0.975)	0.724 (0.713-0.734)	0.856 (0.826-0.887)	0.446 (0.422-0.47)	0.585 (0.561-0.609)
Yes	ADAYSN	Stacking	0.814 (0.804-0.824)	0.436 (0.408-0.463)	0.983 (0.975-0.991)	0.714 (0.695-0.733)	0.923 (0.89-0.955)	0.436 (0.408-0.463)	0.59 (0.565-0.615)
Yes	ADAYSN	AdaBoost	0.813 (0.804-0.822)	0.436 (0.409-0.463)	0.981 (0.974-0.989)	0.732 (0.719-0.745)	0.915 (0.887-0.943)	0.436 (0.409-0.463)	0.589 (0.565-0.613)
Yes	BSMOTE	LR	0.773 (0.761-0.785)	0.445 (0.414-0.476)	0.919 (0.905-0.933)	0.736 (0.721-0.751)	0.714 (0.686-0.741)	0.445 (0.414-0.476)	0.546 (0.52-0.572)
Yes	BSMOTE	RF	0.813 (0.804-0.822)	0.433 (0.409-0.458)	0.983 (0.975-0.991)	0.743 (0.729-0.757)	0.922 (0.893-0.951)	0.433 (0.409-0.458)	0.588 (0.566-0.61)
Yes	BSMOTE	ANN	0.815 (0.805-0.825)	0.441 (0.415-0.467)	0.982 (0.974-0.989)	0.742 (0.724-0.76)	0.918 (0.889-0.947)	0.441 (0.415-0.467)	0.594 (0.57-0.618)
Yes	BSMOTE	SVM	0.816 (0.806-0.825)	0.439 (0.412-0.467)	0.984 (0.977-0.991)	0.749 (0.738-0.761)	0.927 (0.899-0.955)	0.439 (0.412-0.467)	0.594 (0.569-0.619)
Yes	BSMOTE	KNN	0.804 (0.793-0.814)	0.429 (0.403-0.455)	0.971 (0.964-0.978)	0.723 (0.713-0.734)	0.872 (0.846-0.897)	0.429 (0.403-0.455)	0.574 (0.55-0.598)
Yes	BSMOTE	Stacking	0.815 (0.806-0.825)	0.438 (0.412-0.464)	0.984 (0.977-0.99)	0.727 (0.708-0.747)	0.926 (0.9-0.953)	0.438 (0.412-0.464)	0.593 (0.569-0.617)
Yes	BSMOTE	AdaBoost	0.813 (0.804-0.823)	0.436 (0.409-0.462)	0.982 (0.975-0.99)	0.738 (0.723-0.752)	0.919 (0.892-0.947)	0.436 (0.409-0.462)	0.589 (0.566-0.613)
Yes	SMOTEENN	LR	0.795 (0.784-0.805)	0.466 (0.437-0.495)	0.942 (0.93-0.953)	0.735 (0.72-0.75)	0.783 (0.751-0.816)	0.466 (0.437-0.495)	0.582 (0.558-0.607)
Yes	SMOTEENN	RF	0.814 (0.805-0.822)	0.435 (0.409-0.46)	0.983 (0.977-0.989)	0.746 (0.732-0.76)	0.922 (0.898-0.946)	0.435 (0.409-0.46)	0.589 (0.567-0.611)
Yes	SMOTEENN	ANN	0.814 (0.805-0.823)	0.44 (0.414-0.466)	0.981 (0.974-0.988)	0.743 (0.726-0.759)	0.914 (0.888-0.941)	0.44 (0.414-0.466)	0.593 (0.569-0.617)
Yes	SMOTEENN	SVM	0.816 (0.807-0.826)	0.445 (0.417-0.474)	0.982 (0.974-0.99)	0.747 (0.735-0.759)	0.921 (0.891-0.951)	0.445 (0.417-0.474)	0.598 (0.573-0.624)
Yes	SMOTEENN	KNN	0.805 (0.797-0.812)	0.438 (0.418-0.458)	0.968 (0.963-0.974)	0.724 (0.713-0.734)	0.862 (0.843-0.88)	0.438 (0.418-0.458)	0.58 (0.562-0.597)
Yes	SMOTEENN	Stacking	0.816 (0.807-0.825)	0.444 (0.418-0.47)	0.982 (0.976-0.989)	0.711 (0.682-0.74)	0.92 (0.895-0.944)	0.444 (0.418-0.47)	0.597 (0.574-0.621)
Yes	SMOTEENN	AdaBoost	0.815 (0.805-0.824)	0.437 (0.409-0.466)	0.983 (0.977-0.99)	0.735 (0.718-0.752)	0.924 (0.899-0.948)	0.437 (0.409-0.466)	0.591 (0.566-0.617)
